# How Inclusive Interactive Learning Environments Benefit Students Without Special Needs

**DOI:** 10.3389/fpsyg.2021.661427

**Published:** 2021-04-29

**Authors:** Silvia Molina Roldán, Jesús Marauri, Adriana Aubert, Ramon Flecha

**Affiliations:** ^1^Department of Pedagogy, Universitat Rovira i Virgili, Tarragona, Spain; ^2^Faculty of Psychology and Education, University of Deusto, Bilbao, Spain; ^3^Department of Sociology, University of Barcelona, Barcelona, Spain

**Keywords:** interaction, learning, inclusive education, students without special needs, learning environments, interactive groups, dialogic literary gatherings

## Abstract

Growing evidence in recent years has led to an agreement on the importance and benefits that inclusive education has for students with special educational needs (SEN). However, the extension and universalization of an inclusive approach will also be enhanced with more evidence on the benefits that inclusion has for all students, including those without SEN. Based on the existing knowledge that learning interactions among diverse students are a key component of educational inclusion, the aim of this study is to identify the impact on students without SEN of being educated with students with SEN in shared, inclusive, interactive learning environments. Data were collected in three schools using a qualitative approach with a communicative orientation. Semistructured interviews were held with teachers as well as community volunteers participating in the schools. Further, focus groups were conducted with students and teachers. The results show that students without SEN benefit from participating in interactive learning activities with peers with SEN in different ways: (1) they learn to respect others, accept differences, and acknowledge different abilities, thereby creating opportunities for new friendships to develop; (2) they learn about abilities related to helping others participate and learn, to be patient and to gain the satisfaction in helping others learn and behave better; and (3) they benefit from the cognitive effort required to explain themselves and from the contributions of peers with SEN from which they can learn.

## Introduction

The extension and universalization of an inclusive approach is a goal and a challenge for educational systems around the globe, as reflected in the United Nations’ Sustainable Development Goals. Inclusive education means that all children learn together in schools that recognize and respond to the diverse needs of students, ensure quality education for all through appropriate curricula, organization, teaching strategies and resource use (UNESCO, 1994), and overcome the barriers to the presence, participation, and achievement of all students in general education classes (UNESCO, 2017). However, the original idea of inclusive education focuses on the education of a particular group of students—those with special educational needs (SEN)—to overcome practices of special education that have traditionally segregated students based on a medical model of disability (Kurth et al., 2018). In this regard, inclusive education is generally acknowledged as the venue to enhance both the learning and social development of students with disabilities and other SEN, and therefore the way to fulfill their right to shared quality education in mainstream settings (United Nations, 2007). Consequently, discourse, arguments and research about inclusive education have often centered on the collective of students with SEN, and growing evidence has led to an agreement on the benefits that inclusive education has for these students, as found in reviews of recent research. For instance, the meta-analysis conducted by Oh-Young and Filler (2015) compared the outcomes of students with disabilities between placement settings and found that students in more integrated settings outperformed those in more segregated settings, both in the academic and social domains. The recent review of research by Kefallinou et al. (2020) concluded that there is plenty of research that justifies inclusion both from the educational and the social angles, due to the proven positive effects of educational inclusion on the academic outcomes of students with disabilities, and its positive impact on the subsequent social inclusion of people with disabilities in terms of further academic opportunities and qualifications, access to employment and developing personal relationships within the community.

Because inclusive education is about quality education for all, it is important to look at the potential benefits of inclusion for all students. In this regard, the fact that most of the research on inclusive education concerns categories of learners, particularly those with disabilities and other SENs, may cause us to overlook the impacts on other collectives of learners and may not be consistent with a definition of inclusive education geared toward all learners (Messiou, 2017). The objective of extending and universalizing an inclusive approach would benefit from evidence showing that it is positive—or at least not negative—for all students, including those without SEN.

For this reason, some studies have considered the impact of inclusion on students without special needs. Some of these studies have examined the development of students’ attitudes, empathy and understanding of others. For instance, Smith and Williams (2001) showed that children without disabilities can be sensitive to the consequences of different types of impairments and generally have a positive perception of the capabilities of children with different kinds of impairments, which has positive implications for inclusion. Tafa and Manolitsis (2003) found that typically developing children educated in inclusive programs with children with SEN have increased respect, awareness, and acceptance of their peers’ needs, develop less prejudices, and learn to be more helpful and supportive toward people with disabilities, according to parents’ perspectives. This is consistent with other studies that concluded that inclusive education can play a role in challenging disabling attitudes by transforming non-disabled children’s attitudes toward people with disabilities, therefore contributing to building a more inclusive society (Beckett, 2009). Grütter et al. (2017) analyzed the role of friendship between students with and without SEN and found that opportunities to forge close friendships between students with and without SEN enhance the positive attitudes of students without SEN toward students with SEN; this suggests that inclusive education may benefit from educational practices that actively promote friendship among students with and without SEN. Research has also studied the impact of inclusion on the development of cognitive abilities such as theory of mind (ToM), finding that children without SEN educated in inclusive classes with children with SEN develop a better ToM than their peers educated in traditional classes (Smogorzewska et al., 2020). According to Smogorzewska et al. (2020), a greater understanding of diversity, tolerance, acceptance of others and the use of prosocial behaviors in inclusive classrooms seem to promote ToM development.

Other studies have explored the impact on academic learning. Although some studies find that the presence of SEN students in regular classes is related to slightly lower performance of their peers without SEN (e.g., Hienonen et al., 2018), the conclusions of different reviews of research suggest the contrary. Ruijs and Peetsma (2009) revealed that inclusive education has neutral to positive effects for both students with and without SEN compared to non-inclusive education, especially regarding academic achievement. Focusing on the impacts of students without SEN, Kalambouka et al. (2007) showed no evidence of adverse effects of the inclusion of children with SEN, indicating that most findings involved positive or neutral effects on children without SEN. Similarly, Szumski, Smogorzewska and Karwowski’s meta-analysis (2017) underscored a significant and positive—although weak—effect of the presence of students with SEN on the academic achievement of students without SEN. In none of the examined conditions were significant negative impacts found; in contrast, they were at worst neutral and positive in many cases. More recently, Kefallinou et al. (2020) signaled in their review that the inclusion of students with disabilities did not negatively affect the learning outcomes or the social development of their peers without disabilities, and there was a small—but positive—impact on the academic achievement of students without SEN. In addition, the benefits of inclusive education were connected to effective classroom practices characterized by learning interactions, such as cooperative and dialogic learning, peer tutoring, or collaborative problem-solving, which are beneficial for all learners in the classroom (Kefallinou et al., 2020). As argued in these studies, the results support the idea that inclusive education is not against the right of the majority of students to receive quality education, as not only students with SEN, but also those without SEN, may benefit from being educated together.

One of the key characteristics of inclusive educational environments is the opportunity to have rich and diverse learning interactions among heterogeneous students. The role of social interactions in children’s learning and development has long been investigated by psychologists of education since the onset of the sociocultural theory of learning (Vygotsky, 1978; Bruner, 1996). Bruner’s concept of communities of mutual learners helps us to understand the benefits of learning interactions between peers in contexts of diversity. According to Bruner (1996), group work in schools in the form of communities of mutual learners allows for an equilibrium between individuality and group effectiveness, ensuring that everyone progresses according to their ability and giving all children the opportunity “to enter the culture with awareness of what it is about and what one does to cope with it as a participant” (p. 82). Interactive learning spaces, especially when they are mediated by dialogue, permit collective thinking and learning, enhance academic achievement, social skills, and social cohesion, and are especially beneficial for vulnerable groups of students (Fernández-Villardón et al., 2020; García-Carrión et al., 2020). Hence, the objectives of inclusive education would be better attained when such interactive and dialogic learning environments are promoted.

Interactive groups (IGs) and dialogic literary gatherings (DLGs) are specific interactive learning environments that take into account the value of diversity, interaction, and dialogue for learning. Both IGs and DLGs have been identified as successful educational actions (SEAs) that foster successful educational outcomes in diverse student populations (Flecha, 2015). In IGs, classrooms are arranged into small groups of heterogeneous students (e.g., 4–5 students each) who work on instrumental learning activities (especially literacy and math) proposed by the teacher using interaction and dialogue to help each other solve the activity, while a volunteer from the community (e.g., a family member, a former student, or a neighbor) supports each group, dynamizing students’ interactions and mutual help. IGs boost students’ academic learning and—due to the solidary bases of the IG, where students are prompted to help each other—improve the school climate; new friendships are also encouraged, as well as multicultural coexistence (García-Carrión and Díez-Palomar, 2015; Valero et al., 2018; Zubiri-Esnaola et al., 2020).

Dialogic literary gatherings consist of debating books from classical literature that students have previously read. After agreeing to the chapters that will be discussed at the next gathering, students read the text individually or with help from their family members, a teacher, or a peer, and select a piece of text they found relevant to share at the gatherings. There, they discuss and reflect on the text based on the principles of dialogic learning (Flecha, 2000). DLGs contribute not only to a better understanding of the text, but also enhance students’ reading, reasoning, and argumentative abilities, and deepen understanding of others’ perspectives and emotional well-being (García-Carrión, 2015; Garcia et al., 2018; Foncillas et al., 2020).

Both DLGs and IGs have been implemented with students with SEN included in mainstream classrooms, and shared with students without SEN. The interactive learning environments created through IGs and DLGs improve the learning and relationships of students with SEN; therefore IGs and DLGs encompass inclusive learning environments (Duque et al., 2020). Less is known about the impact of IGs and DLGs on students without SEN when they are shared with students with SEN. The aim of this study is to identify impacts for students without SEN of being educated with students with SEN in shared, inclusive, interactive learning environments such as IGs and DLGs.

## Materials and Methods

This research is a qualitative study of schools that implement interactive learning environments—specifically interactive groups (IGs) and dialogic literary gatherings (DLGs)—with students with and without special needs. The study was conducted within the framework of a broader competitive research project titled “Interactive learning environments for the inclusion of students with and without disabilities: Improving learning, development and relationships” (INTER-ACT). More specifically, this study is part of the project’s second objective: “To analyze in depth successful cases of schools implementing IGs and DLGs with students with disabilities to identify the best conditions to increase the impact on the improvement of learning, development, and relationships.”

The specific objectives of this study were: (1) to determine whether participating in IGs and DLGs with students with SEN has an impact in terms of learning and/or development for children without SEN; (2) to identify types of impacts on students without SEN as a result of participating in IGs and DLGs with students with SEN; and (3) to understand how these impacts are related to being educated with students with SEN in shared, inclusive, interactive learning environments such as IGs and DLGs.

### Sample

Data from the three mainstream educational centers that participated in the second objective of the INTER-ACT project were considered. These centers were one primary school, one primary and secondary school, and one secondary school that educate students with and without special needs in shared learning environments, and which have already implemented interactive learning environments (IGs and DLGs) in the framework of an inclusive project. The schools were selected for their participation in the INTER-ACT project according to the following criteria: (a) schools that had been organizing classrooms in IGs and/or DLGs for at least two academic years; (b) these schools serve a higher percentage of students with disabilities than the average in the region; (c) these schools implement IGs and DLGs inclusively, involving students with SEN with their peers who do not have SEN; and (d) these schools had observed improvements in their students, recorded through quantitative or qualitative evidence, since they have implemented IGs and/or DLGs.

### Data Collection

Qualitative data were collected in each school with the aim of understanding, from the participants’ experiences, how the interactive learning environments that were being facilitated with students with and without SEN contributed to students’ cognitive and social development. The data collection techniques used were semistructured interviews with teachers and community volunteers participating in the schools, and focus groups with students and teachers (see Table 1). For the purpose of data collection, students with SEN were considered those with an official report that entailed learning difficulties in the school context. Conversely, students without SEN were those without an official report and who did not present particular learning difficulties in the school context. Purposeful sampling was employed to select participants who could be especially knowledgeable about the object of study. In all cases, the participants selection was agreed with the school principals to select those participants that could be more representative. All data collection techniques were carried out on the school premises for the participant convenience. Interviews with teachers lasted between 60 and 75 min. The duration of the focus groups was approximately 40 min for teachers and between 30 and 45 min for students. In the case of volunteers, interviews lasted approximately 20 min.

**TABLE 1:** Data collection techniques implemented in each school.

	School 1	School 2	School 3	Total
Interviews with teachers	1 Interview (woman)	–	1 Interview (woman)	2 Interviews
Focus groups with teachers	1 FG (4 women)	1 FG (3 women)	–	2 FG
Focus groups with students	2 FG with sixth grade students: Group 1 = 3 boys + 2 girls (1 girl and 1 boy with SEN). Group 2 = 5 girls + 2 boys (1 boy with SEN).	–	1 FG with 2 girls: 1 student of second grade of secondary education with a classmate with SEN. 1 student of third grade of secondary education with SEN.	3 FG
Interviews with community volunteers	–	2 Interviews (men)	–	2 Interviews

Participant teachers in the interviews and in the focus groups were selected based on their experience of implementing IGs and/or DLGs with students with and without SEN. All of them had been implementing IGs and/or DLGs and all of them had—at the moment of the data collection or in the past—students with SEN participating in IGs and/or DLGs together with students without SEN.

Two interviews with teachers were conducted, one in school 1 and one in school 3. They were female teachers in both cases. The teacher interviewed at school 1 was the school principal and a language teacher who implemented DLGs with the two sixth-grade classes, which contained five students with SEN. She had more than 10 years of experience facilitating IGs and DLGs. The teacher interviewed in school 3 taught the third grade of compulsory secondary education. In that class, eight students had SEN.

Two focus groups were held with teachers, one in school 1 and one in school 2. In school 1, four female teachers participated. One of them was a teacher in the first and second grades of primary education, another was a teacher in the third and fourth grades, and two more were teachers in the fifth and sixth grades. They had between 4 and 12 years of experience in the school implementing IGs and/or DLGs. In school 2, three female teachers participated. One of them was a teacher of first and second grade, another was a special education teacher, and the third was a teacher of second grade of compulsory secondary education and educational advisor. They had between 1 and 10 years of experience in the school implementing IGs and/or DLGs.

Three focus groups were held with students, two in school 1 and one in school 3. In school 1, one focus group was conducted with each of the two sixth-grade classes. They have been implementing IGs since second grade and DLGs since third grade. In these classes, cases of special needs included hearing impairment and intellectual disability (one boy), intellectual disability (one boy), dyslexia (two boys and one girl) and ADHD (one boy). Five students participated in the first focus group (three boys and two girls), and seven participated in the second focus group (five girls and two boys). In the first group, there was one girl and one boy with SEN, and in the second group, there was one boy with SEN. In school 3, one focus group was conducted with two girls: one in second grade of compulsory secondary education, and one in third grade of compulsory secondary education. Both participated in IGs and DLGs. One of them had special needs (a syndrome entailing visual and hearing impairment, as well as an intellectual disability) and participated in IGs and DLGs with her classmates without special needs, while the other student did not have SEN and had a classmate with autism who participated in IGs and DLGs along with the rest of the class.

Finally, two interviews were conducted in school 2 with two male volunteers who participated in IGs in classes containing students with and without SEN. One of them had taken part in IGs in preprimary and primary education classes for 2 years, while the other had participated in IGs for 3 years in fifth and sixth grades of primary education and in third grade of compulsory secondary education.

Both the interviews and the focus groups included questions regarding, on the one hand, the characteristics of the implementation of the interactive learning environments and, on the other, the impacts on the participating students. The data collection was conducted using a communicative orientation that involves creating the conditions for egalitarian dialogue between researchers and the end-users of research to reach a shared interpretation of the reality being studied (Gómez et al., 2019). Sample questions for teachers and volunteers included: “How would you describe the interactions between students with SEN and their peers without SEN when they participate in IGs and/or DLGs?” “Have these interactions between students changed over time?” “Have you observed an impact on students that could be related to such interactions?” Sample questions for students were: “How do you work in IGs and DLGs with your classmates?,” “When you or some of your classmates have some difficulties when participating in IGs or DLGs, what do you do?,” “Have you improved on something since you have taken part in IGs and DLGs?,” “And your classmates?,” “Can you give an example?”

Before data collection, school boards and individual participants were informed about the aims of the research. All participants were informed that their participation was voluntary and that the data would be recorded anonymously. Informed consent was obtained from the participant teachers and community volunteers and from the parents or guardians of the minors. To ensure ethical integrity of the study, the research responded to the Universal Declaration of Human Rights adopted by UNESCO, the UN Convention on the Rights of the Child, and the Charter of Fundamental Rights of the EU (2000/C 364/01) regarding scientific and ethical procedures, the European Code of Conduct for Research Integrity (ALLEA, 2017), the Ethics Review Procedure established by the European Commission (2013) for EU research, and the Data Protection Directive 95/46/EC. The study was fully approved by the Ethics Board of the Community of Researchers on Excellence for All (CREA).

### Data Analysis

Interviews and focus groups were audio recorded and transcribed verbatim. Transcriptions were subsequently revised to identify the excerpts that referred to interactions between students with and without SEN that could indicate an impact on students without SEN. A second reading was conducted to identify recurrent themes that emerged from the excerpts, and three main themes were identified that led to the inductive creation of the three categories of analysis: (1) impact on students’ attitudes, (2) impact on students’ social skills, and (3) impact on students’ academic learning and cognitive development (see Table 2). One researcher coded the excerpts according to the categories created; some excerpts were assigned to more than one category. Subsequently, a second researcher revised the coded excerpts, taking into account the definition of the categories. The second researcher agreed on the coding and proposed the assignment of some of the citations to additional categories. The final coding was agreed upon by both researchers.

**TABLE 2:** Categories of analysis.

Category	Definition	Example	Number of quotes	School			Participants		
				1	2	3	T	S	V
1. Impact on students’ attitudes	Evidence regarding the attitudes of students without SEN toward students with SEN, when they learn together in IGs and/or DLGs.	We have built trust with that person for him to understand us and for us to be able to help him even more, so that he overcomes it and he can do it the same as the others do, because no one is better than another one, (…) and that he understands that we support him and we can help him for whatever it is necessary. (Student, school 1)	35 (55%)	17	7	11	26	8	1
2. Impact on students’ social skills	Evidence regarding an impact on the social abilities of students without SEN as a result of learning together with students with SEN in IGs and/or DLGs.	For instance, the other day something very good happened in class, they were writing (…) and one girl already knew that the classmate in front of her was not going to do it well, and said to him—she called him by his name and said— “Remember, ok? Don’t forget that” (…) And it made me smile, because she is a very individualistic girl, but in that moment, she said that spontaneously to take care of him, and I said, ok, good, we have improved. (Teacher, school 1)	27 (42%)	13	11	3	14	9	4
3. Impact on students’ academic learning and cognitive development	Evidence regarding the opportunities for the academic learning and cognitive development of students without SEN when they learn together with students with SEN in IGs and/or DLGs.	And J. explained the meaning of that expression. In addition, it was quite a shock for everyone, and for me, because J., with the difficulties he has in speech, reading, comprehension, everything, was the one who gave the correct explanation; it was quite a shock. (Teacher, school 1)	12 (19%)	11	1	0	9	2	1
TOTAL			64	41	17	14	43	16	5

*T, teachers; S, students; V, volunteers.*

## Results

The results of our analysis allowed us to identify a series of impacts for students without SEN of sharing interactive learning environments with students with SEN. According to the categories of analysis, our findings show that participating together in learning activities, mediated by interaction and dialogue, allows students without SEN to: (1) build understanding and respectful attitudes toward diversity; (2) learn about social abilities related to facilitating others’ learning; and (3) enhance opportunities for academic learning and cognitive development as a result of engaging in learning together, exchanging questions and knowledge. As seen in Table 2, the category with a higher number of quotes is (1) impact on students’ attitudes, with more than half of the quotes referring to such an impact, followed by (2) impact on students’ social skills, and finally by (3) impact on students’ academic learning and cognitive development.

### Building Positive Attitudes Toward Diversity in Interactive Learning Environments Shared With Peers With Special Needs

Category 1 included evidence regarding the attitudes of students without SEN toward students with SEN when they learned together in IGs and/or DLGs. Participants in the three schools, including teachers, students and volunteers, provided evidence in this regard.

When students without SEN share interactive learning environments with students with SEN, they have unique opportunities to learn firsthand about diversity. They share their learning time and space with peers of the same age, who often need special attention because of their individual characteristics, which differ to a greater or lesser extent and in different ways from those of most students. This is a necessary first step to develop positive attitudes on diversity and educational and social inclusion, which cannot be completely achieved when education on respect for diversity, valuing its potential, and educational and social inclusion is not based on the daily experiences of sharing these learning opportunities with individuals with SEN, who have a face and a name. However, interactive learning environments allow students to share not only learning space and time, but also interactions and dialogue around shared learning activities (such as solving a math problem or sharing a personal reflection on an excerpt from a book), which create opportunities to learn about diversity and its value based on the personal experiences of those individuals with whom the activity is shared. In this way, students can learn about diversity with those children who have not only a name and a face but also a personality, preferences, and struggles.

Ana, a secondary education student without SEN who has a classmate with autism spectrum disorder, Jose, explained that getting to know him in the school allowed her to learn about diversity in a way that she could not have done before:

Until I first entered this school last year, I had no idea what the communication and language classroom was, I had no idea that there were people with ASD who could be in schools like this, I was not aware at all of this. However, when I arrived in this school, they put me in the class with Jose, and when I saw him, I said “wow” and I don’t know, from that moment on, he transmitted something to me that made me feel that he was special and that I was going to help him in some way. In addition, as time went by, Jose turned my life around. (Student, school 3)

The interactive learning environment fostered in the classroom, where students learn in dialogue with others, is, according to teachers, what generates the opportunity to acknowledge diversity, while students learn that it is part of human diversity and normalize it:

I believe that it favors inclusion, for sure, because they talk constantly, leaving the classic model of children sitting alone, individually. So yes, they are all integrated. As she said, they always look the same to each other; they do know that one has more difficulties in one thing or another, but they all treat each other equally. (Teachers’ focus group, school 1)

Teachers in the different schools reported a change in attitudes in their students without SEN, who in the interactive learning environments learned about difference, learned to accept it, and to be more respectful about it. Teachers referred, on the one hand, to children’s acknowledgement of individual differences in their peers’ learning process, which became evident as learning activities were shared among the class, either in small interactive groups or in dialogic literary gatherings with the entire class. Students understood that children could learn at different paces and that they can need different kinds of support or adapted materials, but this does not mean that they cannot share the experience of learning; as one teacher explained: “a dynamic of respect and understanding that not everyone does the same has been created” (Teacher, school 1). Importantly, being aware of these differences does not turn into a stigmatization of students with SEN; in contrast, knowing them allows their peers to learn more about their weaknesses, and to better understand their performance in class. The example of shared reading activities illustrates this impact on students’ attitudes:

And the other students, for me this is important, they respect their reading rhythm, they respect it, they know that, depending on which children, they go slowly because they have difficulties, but nobody says so, because we all know that they have difficulties and that they go at their own pace and, if they read it slowly, they understand it well. (Teacher, school 1)

Special needs can be related to areas of curricular learning, but can also be expressed in other ways. Teachers’ experience shows that in interactive learning environments, children learn to be more understanding about other types of difficulties, such as behavioral problems that their classmates may exhibit. Although it may sometimes be annoying, they develop the understanding that these children do not have, at that moment, the ability to behave better and learn to accept it, while teachers work to improve children’s ability to control their behavior. This is the case of what this teacher explained:

There are days when these children—I’m thinking of another one who hasn’t taken the medication—then, he comes in very nervous, he doesn’t stop making noises, he doesn’t shut up. Obviously, holding the gatherings in these conditions is very hard, but they are there, and the group already understand that this child acts this way because he has no other way to do it. Therefore, I think that they have all learned to accept the difference. (Teacher, school 1)

Overall, these episodes show the opportunities created for children without SEN to better understand children with SEN, to be more sensitive to others’ needs, and to be more empathetic. From the perspective of teachers, interactive learning environments such as DLGs entail the learning of values that facilitate the transformation of attitudes. These values emerge from the reading of classic works of literature, which is characteristic of a DLG, where topics such as love, friendship, truth, loyalty, and courage become part of the debate:

In the gatherings many values arise, students work a lot on values and then have a more complete experience, and they share, and they make. They feel empathy for each other. (.) in the classroom it is very difficult for them to put themselves in the other’s place (.) but in the gatherings it isn’t, empathy does come out. (Teacher, school 1)

This learning of values and empathy is also related to the fact that in DLGs, children often link the episodes of reading to episodes about their own lives or other realities they know of. This is how children expressed this idea in their own way:

Because when we give our opinion in the gatherings, sometimes he explains something of his life, and so when he says it, we know slightly more about him, and he says more and more things about his life, and so we get to know each other better and become [better] friends, because in this way we get to know each other much more easily. (Student, school 1)

In this process of knowing their classmates with SEN better as a result of sharing interactive learning environments, children also learn that each individual has different abilities, that all of them may need help at some point, and can help others as well, and that the best learning outcomes are obtained when they share these abilities and help each other. IGs facilitate this process, as in IGs all group members are expected to ensure that all other members understand the activity and complete it; therefore, everyone shares the knowledge and abilities they have and that can contribute to the group work. Teachers in one of the schools reflected on this idea, which also contributed to the change of perceptions and attitudes mentioned, as typically developing students realize that students with SEN have challenges but also have abilities: “In those moments they have truly helped each other. Then, they have realized that it is not always the same people who have to help, but they, who have a challenge, are good at it.” (Teachers’ focus group, school 1)

This acknowledgement of diversity (including difficulties, but also possibilities and diverse abilities), which is due to sharing interactive learning environments, facilitates overcoming prejudices. Students with SEN start to be seen not only as those with poor learning, that always struggle and usually need help, but also as students who are capable of learning and making progress, as one teacher noted:

Academically brilliant boys and girls, who perhaps in third grade looked at these classmates and even knowing them since they were in preschool [3 or 4 years old] thought, “Well, this is clear, they don’t know anything,” have made a positive change because they see these children as classmates with the possibility of learning. (Teacher, school 1)

As shown in this quote from a teacher’s interview, it was not the fact of being educated in the same classroom with SEN students that shaped a realistic perception of their difficulties and capabilities (since both SEN and typically developing students had been educated together for years). Rather the opportunity to learn in interactions with SEN students allowed students without SEN to transform their perceptions and attitudes. Along the same lines, in view of Ana, sharing learning opportunities with her classmate Jose entailed learning that everyone has both difficulties and abilities, and that these can be overcome:

Jose has taught me that many times people have barriers, because we all have barriers, whether it is at the time of learning, at the time of adults finding a job. Whatever, anything, but there is always a way to overcome them, always, and Jose has taught me many things. In fact, I think he has taught me more than I have taught him. (Student, school 3)

This involved shifting the focus from difficulties to possibilities and transforming learning expectations toward them. Importantly, the peer group learned that students with SEN were not only able to learn, but also contributed to the learning of others, which reinforces this change in expectations and the overcoming of prejudices. This might help typically developing students learn to value people not only based on their more evident characteristics—as may be the case with SEN in the school context—but also to pay attention to other traits (which are sometimes hidden) that can give a broader picture of a person and allow for identifying other enriching features. According to teachers, interactive learning environments such as IGs and DLGs permit this to happen:

And from that moment on, I think, that’s when we all realized that children like Javi can participate by making very good contributions, and that girls like Laura don’t know everything. I think that this was a very important moment. (Teacher, school 1)

Further, this greater knowledge of peers with SEN and the development of respect for diversity has led in some cases to the blossoming of new friendships. Ana talked about her special relationship with Jose as something that makes going to school more meaningful for her: “And one of the reasons why I love coming to school is to have Jose’s smile there every morning (.) and it’s something I wouldn’t change for anything in the world” (Student, school 3). Blanca, a girl with SEN in the same secondary school, explained something similar in terms of when she thinks of her classmate and friend Jaume:

Like Ana said, she is very happy with Jose. I am exactly the same with Jaume (.) I am very happy with him and I am happy to have him as a friend, and he is special and very important to me. (Student, school 3)

The building of these friendships not only has had an impact within the school, but has also transferred and expanded the benefits of interactions between students with and without disabilities to new contexts outside school premises and across time, as a teacher in that school explained:

[His] friendship within the school [was] prolonged on weekends (.) He has come to meet [his] friends of the classroom to go out to dinner 1 day, to see a movie and that is very interesting (.) I think the fact of having worked in groups has facilitated doing things, not only in his group of six, because these groups have been changing more or less. (Teacher, school 3)

### Learning Social Skills Related to Helping Others Participate and Learn

Category 2 included evidence regarding an impact on the social abilities of students without SEN as a result of learning together with students with SEN in IGs and/or DLGs. Participants in the three schools, including teachers, students and volunteers, offered evidence in this regard.

In addition to the transformation of thoughts, attitudes and the acknowledgment of others’ abilities and difficulties, engaging in learning interactions with peers with SEN helps to develop a series of social skills. Children acquire these skills because they are necessary to interact with their classmates in IGs and DLGs, specially with those with SEN. These interactive learning environments pose this demand, and these skills become part of the repertoire of abilities that children can use in multiple contexts and with diverse people. First, in interactive learning environments such as IGs and DLGs, children are expected to help each other; thus, children progressively get used to and develop this ability to support their peers, as well as receiving help when necessary. Both teachers and volunteers reflected on the way children learned about this ability through time: “Last year I did notice a change, yes (.) in the end they learn to collaborate, above all, to help each other, and that it goes well, and the work comes out, which is what we are looking for.” (Volunteer, school 2)

With the practice of helping each other in interactive and diverse learning environments, children come to see that collaboration among all helps everyone’s learning, as it allows for one to take advantage of the diverse abilities in the group; therefore, they become progressively more motivated and more proficient in this activity:

Everyone has some skills; some have some skills for one thing and others have some skills and some abilities for another. After all, if there is a collaboration between all, it is where you have to reach an end, and they help each other to reach this end. (Teacher, school 2)

Once they acquire this ability, they use it to help anyone who needs it, including children with more learning difficulties; they normalize helping others and realize they can make a difference in the learning opportunities of the students with the most difficulties. Therefore, and as a volunteer explained, all students in her class were willing to help those who were more in need: “Yes, let’s say, the whole group was dedicated to helping them” (Volunteer, school 2). Consequently, when they share learning activities with students who especially struggle with learning, they find the opportunity to strengthen this ability to help. Blanca explained something similar when not just one, but three classmates went to help her with the activity:

For example, in History, we also do [interactive] groups. We were doing a mapping exercise and (.) I got lost a little bit, then I asked my classmate sitting next to me to help me and so on, then she came to help me, then two more came to help me, and I was happy because I did not make myself clear, I got nervous, I did not know how to do it, then (.) they came to help me (.), and that is the best thing about being in a group. (Student, school 3)

Second, in this attempt to help their peers with SEN and facilitate their participation in interactive learning environments, they learn to adjust their interactions to the particular needs of each child. For instance, they learn to be patient and to give the necessary time when their peers have a slower learning pace, which is an evidence of the empathy developed:

In the gatherings they have also learned to give time. For example, a girl I have in class has a hard time explaining herself, but in the end, she gets it out. Therefore, they have learned to be patient with her and not to stand up and let her talk. Then, in the end, they realize that she does, that she gets out, that she explains well. (Teacher, school 1)

In this regard, they learn to provide adjusted support, building on the abilities they acknowledge in these peers, and try to find alternative ways so that these children can participate in the activity. This entails a metacognitive effort when they try to understand what these children know and how they can help them participate in the activity and progress in their learning.

The atmosphere in the classroom, when there is a group with a child with SEN, the others, as they live it in their daily life, apart from understanding the difficulty he has and stay on their level, they also look for ways in which he can participate and get involved in some way in the activity. (Teacher, school 1)

This effort to facilitate the learning and participation of children with SEN becomes part of the class routine. so as the teachers explained, it unites the group around this shared purpose and the group members become more sensitive to the needs of their peers. This is also achieved thanks to the guidance that teachers and volunteers provide in order to help typically developing students adjust the support they offer to their SEN peers, and also to encourage typically developing students to help their SEN peers while avoiding overprotection:

In other words, their classmates, or at least what I experience from my class, they are very supportive and, as Maria said, they are very sensitive on this subject. In this case, I have two students [with SEN], and they take care of them, not too much, because they must be reminded to let them think, too. However, they do take them very much into account in regard to working in [interactive] groups. They try to make sure they can participate like everyone else. Of course, within their possibilities. (Teacher, school 1)

As a result, the situations created not only turn into a higher ability to help others, but also in the satisfaction of seeing others learn better due to their help, which reinforces this behavior. Teachers noted this impact on children: “They help each other and it is going very well; and they love it, it is something they like very much” (Teacher, school 2), as well as students themselves: “And, when you help him and you see that he understood it, you feel satisfied” (Student, school 2). “When I help Joan or even when Joan helps me more, I feel more fulfilled with myself, happier” (Student, school 3). Such rewarding experiences motivates them to continue participating in these activities and to help others, which benefits everyone’s learning.

### Enhancing the Opportunities for Academic Learning and Cognitive Development

Category 3 included evidence regarding opportunities for the academic learning and cognitive development of students without SEN when they learned together with students with SEN in IGs and/or DLGs. Participants in school 1 and school 2, including teachers, students and volunteers, mentioned this type of impact.

Sharing learning activities with students with SEN in interactive learning environments triggers an additional cognitive effort for typically developing children when they try to explain themselves to their peers with SEN. It entails, on the one hand, putting oneself in the other’s shoes, trying to understand his/her difficulties and thinking of how to help him/her overcome these difficulties, thus gaining from the cognitive effort made and reinforcing their learning. On the other hand, it also entails discovering one’s own difficulties when trying to make oneself be understood and to do one’s best to achieve it. In this regard, such situations allow students who do not usually have learning challenges to experience them, and underscore the need to make an effort to achieve their objective, which contributes to being more empathetic and understanding of their peers with SEN and, sometimes, humbler regarding their own abilities, as one volunteer explained:

They do this effort of trying to make them be understood by the other, and this is very interesting, as the know-it-all can see his/her own limitations with respect to the others. Therefore, it demands a much greater effort from oneself than usual. (Volunteer, school 2)

In addition, in interactive learning environments, students without SEN can learn from the explanations and contributions of children with SEN. IGs and DLGs are characterized by promoting a framework of open and egalitarian dialogue where all contributions are valued based on validity claims (i.e., the value of the contribution’s content, regardless of who made the contribution, and in this case, regardless of whether it is a student with or without SEN). Learning from students with SEN can occur both in IGs and in DLGs when these students have a good understanding of the concepts they are working on. As noted by one teacher, these episodes are opportunities for the entire group to learn:

Children with many special difficulties, have been the ones who have given the clarification, the definition, the explanation for the rest of the group to understand, and this has created a situation, which is not seen, but it is noticed, of improvement for all. (Teacher, school 1)

In DLGs, it also occurs when children with SEN share the paragraph or idea they selected to bring to the gathering, or when they raise doubts about the meaning of particular words that other students had not paid attention to—although they might not understand it either—and this opens up a debate on the meaning of that word or on the ideas of that paragraph that may have not existed without the participation of these children. In the following quote from a teacher, we find first a reference to those situations when a child with SEN does not understand something and their peers explain it to him/her, provoking the additional cognitive effort of trying to make something be understood. Next, we find the reference to these other situations when children with SEN contribute to the group bringing their questions, doubts, and interventions to the gathering, opening a learning opportunity for all:

If they do not understand it, their classmates explain the meaning to them. Then, when we do this rereading of the chapter or the pages, other vocabulary words often appear that, perhaps nobody had chosen or they do not know the meaning of, and then another debate starts about knowing what it means. Or someone raises their hand and says, “I had not chosen this because when I read it perhaps it did not catch my attention, but now when I reread the chapter, I want to comment on it,” and right after it is commented on. This is done both by children with SEN and by the rest of the class, regardless of their level of ability and everything else. A climate is created that is similar to magic. (Teacher, school 1)

According to the participants’ experiences, interactive learning environments shared between students with and without SEN create the opportunity for all to acknowledge that everyone has abilities and difficulties. Children with SEN can surprise others with their questions, responses, and contributions, generating new opportunities for learning, and everyone can learn that children without SEN do not always know everything. As one teacher explained based on her experience over the years, the fact that children with SEN share interactive learning environments with their peers without SEN has not only benefitted these SEN children, but also the dynamics of the classroom, as it is enriched with diversity, and therefore becomes a benefit for all:

The fact that these children are in the group—and I can talk about it already for the past 4 years—has improved the dynamics of the gatherings. I think it has been beneficial for everyone, and I am sure it has, because they make interventions that even they themselves are often surprised to have made, and their peers have seen this. (Teacher, school 1)

## Discussion

Interactive groups and DLGs are interactive learning environments that have already been demonstrated to be inclusive and lead to positive academic and social impacts for students with SEN (Duque et al., 2020). The study presented here is the first to analyze the potential impacts of IGs and DLGs on students without SEN when they share these interactive learning environments with students with SEN. The results of our study show that students without SEN can benefit from participating in interactive learning environments (such as IGs and DLGs) with peers with SEN in at least three different ways: (1) building positive attitudes as they learn to respect others, accept differences, and acknowledge different abilities, creating opportunities for new friendships; (2) enhancing their social skills, as they learn about abilities related to helping others participate and learn, to be patient, and gain satisfaction from helping others learn; and (3) producing opportunities to enhance academic learning and foster cognitive development, as they gain from the cognitive effort needed to explain themselves and from the contributions of peers with SEN from which they can learn. Importantly, we did not find negative impacts for students without SEN or for those with SEN as a result of sharing these interactive learning environments. In contrast, all impacts identified—either at the attitudinal, social, or cognitive level—were positive for both groups of students.

In the cases studied, children without SEN developed positive attitudes toward diversity in IGs and DLGs. This is in the line of previous research which found that inclusive educational environments are related to more positive attitudes toward diversity, and especially more positive attitudes among typically developing peers toward children with disabilities or other SEN (Smith and Williams, 2001; Beckett, 2009). It is also consistent with research that found that solidarity can be learned in the school context and that it contributes to creating genuine attitudes of inclusion beyond the norms that benefit everyone (Hernández Arteaga et al., 2020).

Additionally, we found that students without SEN had the opportunity to develop social skills when they learned together with students with SEN in IGs and DLGs. Identifying particular types of classroom arrangements and learning dynamics (such as IGs and DLGs) that help one to cultivate such attitudes and skills is important not only for students with SEN—who are more respected, accepted, and integrated in their group of peers—but also beneficial for students without SEN. Attitudes of understanding diverse identities; the values of justice, equality, dignity and respect; cognitive skills (including the ability to adopt a multiperspective approach); social skills (such as empathy and conflict resolution), communication skills and aptitudes for interacting with diverse people, and the capacity to act collaboratively and responsibly have been highlighted as key competences necessary in the 21st century (UNESCO, 2014).

Moreover, we found a positive impact of the interactive learning environments created with IGs and DLGs on opportunities for the learning and cognitive development of children without SEN. This is in line with previous research comparing the learning outcomes of students without SEN, who are educated with students with SEN, and those who are not, which overall revealed no negative impacts on these students but, on the contrary, positive impacts or neutral in the worst cases (Kalambouka et al., 2007; Ruijs and Peetsma, 2009; Szumski et al., 2017; Kefallinou et al., 2020).

These findings should be taken cautiously. On the one hand, because the study is based on a reduced sample, the conclusions cannot be generalized. On the other hand, because data were collected in schools that were already implementing IGs and DLGs, a pre-post intervention comparison cannot be made to ascertain the changes that occurred in students without SEN due to sharing IGs and DLGs with students with SEN. Finally, the qualitative nature of the data facilitates an understanding of the reality studied but does not allow for a precise assessment of the impacts on students without SEN. Subsequent research could expand the analysis to a broader sample and include an examination of quantitative data, especially of students’ academic progress, since the third category of analysis (impact on students’ academic learning and cognitive development) is the one for which we obtained the least evidence.

However, as the first study on this topic, this research enables an initial approximation based on the participants’ experiences, which is consistent with previous knowledge and can be the basis for further investigation. First, it is in line with the results of previous research on DLGs and IGs which shows their impact on improving students’ academic learning, a better understanding of others and positive coexistence (García-Carrión, 2015; García-Carrión and Díez-Palomar, 2015; Garcia et al., 2018; Valero et al., 2018; Foncillas et al., 2020; Zubiri-Esnaola et al., 2020). Our study suggests that sharing IGs and DLGs with students with SEN creates new conditions in which these improvements can be promoted. Second, it is aligned with past research on inclusion, which has associated the benefits of inclusive education with classroom practices characterized by interaction, dialogue, and collaboration (Kefallinou et al., 2020), all of which are characteristics of IGs and DLGs and could thus explain the benefits observed. Third, it is in line with theoretical contributions that refer to the relevant role of peer help and other forms of sharing learning interactions. When children try to explain learning content to their peers with SEN or try to help them solve a problem, they expand what Vygotsky called the zone of proximal development (1978) or what Bruner called scaffolding (1996). Both authors emphasized (stemming from the sociocultural theory of learning) the importance of interactions for children’s learning and argued that these interactions could emerge not only from adults but also from more capable peers. Interactions allow for the creation of shared learning (Mercer and Littleton, 2007), and our data indicate that more capable peers can also benefit from these interactions and find opportunities to advance their learning and cognitive development. Indeed, research has suggested thinking of the zone of proximal development not in terms of knowledge transmission, but as an encounter of consciousness that mutually benefits the participants in the interaction (Roth and Radford, 2010).

Although further research is necessary to have a more precise description of the impact of IGs and DLGs for students without SEN when they share these learning environments with students with SEN, the evidence presented can contribute to the understanding that inclusive education not only benefits the most vulnerable students (such as students with disabilities and other SENs), but can also benefit all students when interactions and dialogue are promoted in contexts of diversity. Therefore, it is the right of everyone—with or without SEN—to be educated in inclusive, interactive learning environments, as they produce unique conditions for the academic and human development of all students.

## Data Availability Statement

The raw data supporting the conclusions of this article will be made available by the authors, without undue reservation.

## Ethics Statement

The studies involving human participants were reviewed and approved by the Ethics Board of the Community of Researchers on Excellence for All (CREA). Written informed consent to participate in this study was provided by the participants’ legal guardian/next of kin.

## Author Contributions

RF conceptualized the research. SM conducted the literature review, a preliminary analysis of the data, and a first draft of the manuscript. JM revised the data analysis. RF, AA, and JM revised the manuscript and provided feedback and corrections. SM revised the final version of the manuscript. All authors contributed to the article and approved the submitted version.

## Conflict of Interest

The authors declare that the research was conducted in the absence of any commercial or financial relationships that could be construed as a potential conflict of interest.

## References

[B1] ALLEA (2017). *The European Code of Conduct for Research Integrity.* Available online at: https://www.allea.org/wp-content/uploads/2017/05/ALLEA-European-Code-of-Conduct-for-Research-Integrity-2017.pdf [accessed January 5, 2021]

[B2] Beckett A. E. (2009). Challenging disabling attitudes, building an inclusive society: considering the role of education in encouraging non-disabled children to develop positive attitudes towards disabled people. *Br. J. Sociol. Educ.* 30 317–329. 10.1080/01425690902812596

[B3] Bruner J. (1996). *The Culture of Education.* Cambridge, MA: Harvard University Press.

[B4] Duque E. Gairal R. Molina S. Roca E. (2020). How psychology of education contributes to research with social impact on the education of students with special needs: the case of successful educational actions. *Front. Psychol.* 11:439. 10.3389/fpsyg.2020.00439 PMC7082399 32231627

[B5] European Commission (2013). *Ethics for Researchers. Facilitating Research Excellence in FP7.* Available online at: http://ec.europa.eu/research/participants/data/ref/fp7/89888/ethics-for-researchers_en.pdf [accessed January 5, 2021]

[B6] Fernández-Villardón A. Álvarez P. Ugalde L. Tellado I. (2020). Fostering the social development of children with special educational needs or disabilities (send) through dialogue and interaction: a literature review. *Soc. Sci.* 9:97. 10.3390/socsci9060097

[B7] Flecha R. (2000). *Sharing Words: Theory and Practice of Dialogic Learning.* Lanham, M.D: Rowman & Littlefield.

[B8] Flecha R. (2015). *Successful Educational Action for Inclusion and Social Cohesion in Europe.* Berlin: Springer.

[B9] Foncillas M. Santiago-Garabieta M. Tellado I. (2020). Análisis de las tertulias literarias dialógicas en educación primaria: un estudio de caso a través de las voces y dibujos argumentados del alumnado. *Multidisciplinary J. Educ. Res.* 10 205–225. 10.17583/remie.2020.5645

[B10] Garcia C. Gairal R. Munté A. Plaja T. (2018). Dialogic literary gatherings and out-of-home child care: creation of new meanings through classic literature. *Child Fam. Soc. Work* 23 62–70. 10.1111/cfs.12384

[B11] García-Carrión R. (2015). What the dialogic literary gatherings did for me. *Qualitative Inquiry* 21 913–919. 10.1177/1077800415614305

[B12] García-Carrión R. Díez-Palomar J. (2015). Learning communities: pathways for educational success and social transformation through interactive groups in mathematics. *Eur. Educ. Res. J.* 14 151–166. 10.1177/1474904115571793

[B13] García-Carrión R. López, de Aguileta G. Padrós M. Ramis-Salas M. (2020). Implications for social impact of dialogic teaching and learning. *Front. Psychol.* 11:140. 10.3389/fpsyg.2020.00140 PMC7012899 32116941

[B14] Gómez A. Padrós M. Ríos O. Mara L. C. Pukepuke T. (2019). Reaching social impact through communicative methodology. researching with rather than on vulnerable populations: the roma case. *Front. Educ.* 4:9.

[B15] Grütter J. Gasser L. Malti T. (2017). The role of cross-group friendship and emotions in adolescents’ attitudes towards inclusion. *Res. Dev. Disabil.* 62 137–147. 10.1016/j.ridd.2017.01.004 28160623

[B16] Hernández Arteaga I. Fernández López K. M. Estela Vasquez A. C. Mestizo Nuzcue E. J. (2020). Educación y solidaridad: un camino hacia la inclusión educativa. *Soc. Educ. History* 9 227–251.

[B17] Hienonen N. Lintuvuori M. Jahnukainen M. Hotulainen R. Vainikainen M. P. (2018). The effect of class composition on cross-curricular competences – Students with special educational needs in regular classes in lower secondary education. *Learn. Instruction* 58 80–87. 10.1016/j.learninstruc.2018.05.005

[B18] Kalambouka A. Farrell P. Dyson A. Kaplan I. (2007). The impact of placing pupils with special educational needs in mainstream schools on the achievement of their peers. *Educ. Res.* 49 365–382. 10.1080/00131880701717222

[B19] Kefallinou A. Symeonidou S. Meijer C. J. W. (2020). Understanding the value of inclusive education and its implementation: a review of the literature. *Prospects* 49 135–152. 10.1007/s11125-020-09500-2

[B20] Kurth J. A. Miller A. L. Toews S. G. Thompson J. R. Cortés M. Dahal M. H. (2018). Inclusive education: perspectives on implementation and practice from international experts. *Intellect. Dev. Disabil.* 56 471–485.

[B21] Mercer N. Littleton K. (2007). *Dialogue and the Development of Children’s Thinking, a Socio-Cultural Approach.* Milton Park: Routledge.

[B22] Messiou K. (2017). Research in the field of inclusive education: time for a rethink? *Int. J. Inclusive Educ.* 21 146–159. 10.1080/13603116.2016.1223184

[B23] Oh-Young C. Filler J. (2015). A meta-analysis of the effects of placement on academic and social skill outcome measures of students with disabilities. *Res. Dev. Disabil.* 47 80–92. 10.1016/j.ridd.2015.08.014 26342328

[B24] Roth W. M. Radford L. (2010). Re/thinking the zone of proximal development (Symmetrically). *Mind Cult. Act.* 17 299–307. 10.1080/10749031003775038

[B25] Ruijs N. M. Peetsma T. T. D. (2009). Effects of inclusion on students with and without special educational needs reviewed. *Educ. Res. Rev.* 4 67–79. 10.1016/j.edurev.2009.02.002

[B26] Smith L. A. Williams J. M. (2001). Children’s understanding of the physicals cognitive and social consequences of impairments. *Child Care Health Dev.* 27 603–617. 10.1046/j.1365-2214.2001.00236.x 11737026

[B27] Smogorzewska J. Szumski G. Grygiel P. (2020). Theory of mind goes to school: does educational environment influence the development of theory of mind in middle childhood? *PLoS One* 15:e0237524. 10.1371/journal.pone.0237524 32797114 PMC7428351

[B28] Szumski G. Smogorzewska J. Karwowski M. (2017). Academic achievement of students without special educational needs in inclusive classrooms: a meta-analysis. *Educ. Res. Rev.* 21 33–54. 10.1016/j.edurev.2017.02.004

[B29] Tafa E. Manolitsis G. (2003). Attitudes of Greek parents of typically developing kindergarten children towards inclusive education. *Eur. J. Special Needs Educ.* 18 155–171. 10.1080/0885625032000078952

[B30] UNESCO (1994). *The Salamanca Statement and Framework for action on special needs education: Adopted by the World Conference on Special Needs Education, Access and Quality.* Paris: UNESCO.

[B31] UNESCO (2014). *Global Citizenship Education. Preparing learners for the challenges of the 21st century.* Paris: UNESCO.

[B32] UNESCO (2017). *A Guide for Ensuring Inclusion and Equity in Education.* Paris: UNESCO.

[B33] United Nations (2007). *Convention on the Rights of Persons with Disabilities (CRPD).* Available online at: https://www.un.org/development/desa/disabilities/convention-on-the-rights-of-persons-with-disabilities.html [accessed January 5, 2021]

[B34] Valero D. Redondo-Sama G. Elboj C. (2018). Interactive groups for immigrant students: a factor for success in the path of immigrant students. *Int. J. Inclusive Educ.* 22 787–802. 10.1080/13603116.2017.1408712

[B35] Vygotsky L. S. (1978). *Mind in Society: the Development of Higher Psychological Processes.* Boston: Harvard University Press.

[B36] Zubiri-Esnaola H. Vidu A. Rios-Gonzalez O. Morla-Folch T. (2020). Inclusivity, participation and collaboration: learning in interactive groups. *Educ. Res.* 62 162–180. 10.1080/00131881.2020.1755605

